# Gastroprotective effect of carob (*Ceratonia siliqua* L.) against ethanol-induced oxidative stress in rat

**DOI:** 10.1186/s12906-015-0819-9

**Published:** 2015-08-20

**Authors:** Kaïs Rtibi, Mohamed Amine Jabri, Slimen Selmi, Abdelaziz Souli, Hichem Sebai, Jamel El-Benna, Mohamed Amri, Lamjed Marzouki

**Affiliations:** Laboratoire de Neurophysiologie Fonctionnelle et Pathologies, Département des Sciences Biologiques, Faculté des Sciences de Tunis. Campus Universitaire El Manar II, Tunis, 2092 Tunisia; Laboratoire de Physiologie Fonctionnelle et Valorisation des Bio-Resssources, Institut Supérieur de Biotechnologie de Béja, Université de Jendouba, Avenue Habib Bourguiba, B.P, 382-9000, Béja, Tunisia; Laboratoire de Physiologie Intégrée, Faculté des Sciences de Bizerte, 7021 Zarzouna, Bizerte, Tunisia; INSERM, U1149, Centre de Recherche Sur l’Inflammation, Faculté de Médecine X. Bichat, 75018 Paris, France

**Keywords:** Gastric ulcer, Carob pods, Oxidative stress, Antioxidant capacity, Rat

## Abstract

**Background:**

We aimed in the present study, at investigating the gastroprotective effect of carob pods aqueous extract (CPAE) against ethanol-induced oxidative stress in rats as well as the mechanism implicated.

**Methods:**

Adult male wistar rats were used and divided into six groups of ten each: control, EtOH (80 % v/v, 4 g/kg *b.w.*), EtOH 80 % + various doses of CPAE (500, 1000 and 2000 mg/kg, *b.w.*) and EtOH + Famotidine (10 mg/kg, p.o.) Animals were perorally (*p.o.*) pre-treated with CPAE during 15 days and intoxicated with a single oral administration of EtOH (4 g/kg *b.w.*) for two hours.

**Results:**

The colorimetric analysis demonstrated that the CPAE exhibited an importance *in vitro* antioxidant activity against ABTS and DPPH radicals. We found that CPAE pretreatment *in vivo*, protected against EtOH-induced macroscopic and histological changes induced in stomach mucosa. Carob extract administration also protected against alcohol-induced volume gastric juice decrease. More importantly, We showed that CPAE counteracted EtOH-induced gastric lipoperoxidation, reversed the decrease of sulfhydryl groups (−SH) an hydrogen peroxide (H_2_O_2_) levels, and prevented the depletion of antioxidant enzyme activity of superoxide dismutase (SOD), catalase (CAT) and glutathione peroxidase (GPx).

**Conclusions:**

These findings suggest that CPAE exerted a potential gastro-protective effect against EtOH-induced oxidative stress in rats, due in part, to its antioxidants properties.

## Background

In gastrointestinal disorders, ulcer is a common disease with multiple etiologies. This disease, characterized by mucosal damage, is predominantly caused by *Helicobacter pylori*, antiplatelet agents such as acetylsalicylic acid [1], nonsteroidal anti-inflammatory drugs (NSAIDs) such as oral bisphosphonates, potassium chloride, immunosuppressive medications [2, 3], serotonin reuptake inhibitors [4], cigarette smoking and alcohol consumption [5]. The pathophysiology of gastric ulcer has generally focused on imbalance between aggressive and protective factors in the stomach, such as acid-pepsin secretion, mucosal barrier, mucus secretion, blood flow, cellular regeneration, prostaglandins and epidermal growth factors [6]. Ethanol-induced gastric lesions is mainly related to intense infiltration in the sub-mucosa that promotes formation of reactive oxygen species (ROS), decreased mucus, depletion of sulfhydryl groups and decreased blood flow, resulting in damage of the gastric mucosa [7]. ROS especially hydroxyl radical play the major role in causing oxidative damage of mucosa in all types of ulcers [8]. To determine the possible mechanism by which substances can act to promote gastroprotection, several antioxidants molecules such as quercetin [9] and curcumin [10] were previously investigated.

Carob (*Ceratonia siliqua L.*) is a slow-growth ever-green tree cultivated for years in Mediterranean countries. The carob fruit, brown pod 10–25 cm in length, contain many bioactive substances such as sweet carbohydrate, dietary fiber, tannins, and polyphenols [11]. Many of the beneficial health effects associated to consumption of phenolic-rich foods are essentially due to their antioxidant activities [12, 13]. For carob extract, this property has been previously reported in *in vivo* and *in vitro* studies [14, 15]. Recently, we and other discovered that Tunisian leaf carob extract presents some ameliorative effects against alcohol or CCl_4_-induced oxidative damage in rats tissues [16, 17]. In addition, carob fiber exhibit high antioxidant capacity determined by the DPPH radical scavenging test, i.e., higher than many other foods rich in polyphenols, such as blueberries, grapes or red wine [18, 19].

Accordingly, the present study was designed to evaluate the putative gastroprotective role of the aqueous extract of carob pods (CPAE) (15 days) against oxidative stress induced by acute ethanol exposure and the mechanism involved in such protection. 

## Methods

### Ethics statement

The necessary permits for the field studies and collection of carob pods samples were obtained by the Ministry of Agriculture in Tunisia and identified by Mrs. Mouhiba Ben-Naceur, professor of taxonomy in the Higher Institute of Biotechnology of Béja, Tunisia. The Voucher specimens have been deposited with the herbarium of the Higher Institute of Biotechnology of Béja and also in the Department of Biological Sciences, Faculty of Science, Tunisia.

### Preparation of carob extract

The mature carob pods were collected from the region of Tabarka (North-West of Tunisia) during October 2013. Briefly, the plant material was later dried in an incubator at 50 °C during 72 h and powdered in an electric blender (Moulinex Ovatio 2, FR). Powder mixture containing carob pulp (90 %) and seeds (10 %) was dissolved in double distilled water and filtered through a colander (0.5 mm mesh size). Finally, the carob pods aqueous extract was immediately used for *in vitro* and *in vivo* experiments.

### Free radical-scavenging activities on DPPH

The antioxidant capacity of the aqueous extract of carob pods was performed using 2,2-diphenyl-1-picrylhydrazyl (DPPH) radical-scavenging activity as previously described by Grzegorczyk et al. [20]. Briefly, various concentrations of CPAE (20, 50, 100, 150, and 200 μg/ml) were added to 1 ml of 0.1 mM methanol solution of DPPH and incubated at 27 °C during 30 min. The optical density of the sample was quantified at 517 nm. DPPH radical-scavenging activity (RSA), expressed as a percentage, was estimated utilizing the following formula:

$$ \mathrm{R}\mathrm{S}\mathrm{A}\left(\%\right)=\frac{{{{}^A}_{\mathrm{DPPH}}}^{-}\left({{}^{A_{\mathrm{sample}}}}^{-{A}_{\mathrm{control}}}\right)}{{}^{A_{\mathrm{DPPH}}}}\times 100 $$

Ascorbic acid was used as a reference molecule in the same concentration as the test extract.

All the analyses were executed in triplicate. The efficacy concentration 50 (EC_50_) value was determined as the concentration (in μg/ml) of the compound required to scavenge 50 % of the DPPH radical.

### Free radical-scavenging activities on ABTS

The antioxidant capacities of the carob pods aqueous extract were evaluated using the 2,2’-azino-bis [3-ethylbenzthiazoline-6-sulphonic acid] (ABTS) method [21]. Briefly, 1 ml of diluted extract was added to 3 ml of 7 mM ABTS radical solution (ABTS•+) and was kept in dark at room temperature for 60 min. The absorbance was measured at 734 nm. The scavenging capacity was calculated as ((1 − Ab/A0) × 100 %) (Ab and A0 are the absorbance of samples as well as the ABTS^•+^ solution at 734 nm.

## Animals and treatment

Healthy adult male Wistar rats (weighing 220–250 g; housed five per cage) and adult male Swiss Albino mice (weighing approximately 25 g; housed ten per cage) were purchased from Society of Pharmaceutical Industries of Tunisia (SIPHAT, Ben-Arours, TN). Experimental protocols were approved with the guidelines of the Ethical Committee of Science Faculty of Tunis, Tunisia. The test was performed in compliance with the Commission Directive 2000/32/EC and the OECD Guideline 474 [22]. They were provided with standard food (standard pellet diet- Badr Utique-TN) and water *ad libitum* and maintained in animal house at controlled temperature (22 ± 2 °C) with a 12 h light–dark cycle. The rats were divided into half a dozen groups. Group 1 and 2 were served as controls and had bidistilled water (5 mL/kg, *b.w.*, *p.o.*). Groups 3, 4, and 5 were pre-treated with various doses of CPAE (500, 1000 and 2000 mg/kg, *b.w. p.o.*), while group 6 was pre-treated respectively with famotidine (10 mg/kg, *b.w. p.o.*) during 15 days. Rats were fasted for 24 h before the last administration of CPAE or reference molecules. After 60 min, each animal, except group 1, received EtOH (4 g/kg, *b.w.*) by oral administration. Two hours later, rats were sacrificed.

### Acute toxicity study

The carob pods aqueous extract in the dose range of 0.05, 0.1, 0.5, 1, 2, 5, 10 and 200 g/kg was orally administrated to different groups of mice (*n* = 10). The animals were examined every 30 min during 4 h and then, occasionally for additional period of 8 h. 24 h after, the mortality was recorded. The mice were also observed for other signs of toxicity, such as motor co-ordination, righting reflex and respiratory changes.

### Evaluation of gastric mucosal damage

The stomach of each animal was removed and opened along its greater curvature. The tissue was gently rinsed in NaCl 0.9 %. The lesions in the gastric mucosa were macroscopically examined and the photographs of hemorrhagic erosions were acquired with a Photometrics Quantix digital camera. Ulcer indexes were determined as the sum of the lengths of the whole gastric lesions (in mm^2^) [23]. Two independent, blinded observers performed the measurements of lesion lengths.

### Gastric volume juice determination

Gastric juice was collected and centrifuged at 3000 g during 5 min to remove insoluble materials. The supernatant was after measured using graduate tubes [24].

### Histopathological analysis

Immediately after sacrifice, small pieces of stomach were harvested and washed with icecold saline. Tissue fragments were then fixed in a 10 % neutral buffered formalin solution, embedded in paraffin and used for histopathological examination. 5 μm thick sections were cut, deparaffinized, hydrated and stained with hematoxylin and eosin (HE). The gastric sections were examined in blind fashion in all treatments [25].

### Lipid peroxidation measurement

Gastric mucosa lipid peroxidation was determined by MDA measurement according to the double heating method [26]. Briefly, aliquots from gastric mucosa homogenates were mixed with BHT-trichloroacetic acid (TCA) solution containing 1 % BHT (*w/v*) dissolved in 20 % TCA (*w/v*) and centrifuged at 1000 × *g* for 5 min at 4 °C. Supernatant was blended with a solution containing (0.5 N HCl, 120 mM TBA buffered in 26 mM Tris) and then heated at 80 °C for 10 min. After cooling, the absorbance of the resulting chromophore was determined at 532 nm. MDA levels were determined by using an extinction coefficient for MDA-TBA complex of 1.56 × 10^5^ M^−1^ · cm^−1^.

### Thiol group measurement

The total concentration of thiol groups (−SH) was performed according to Ellman’s method [27]. Briefly, aliquots from gastric mucosa were mixed with 100 μl of 10 % SDS and 800 μl of 10 mM phosphate buffer (pH 8), and the optical density was measured at 412 nm (A_0_). After adding 100 μl of 5,5’-dithiobis-(2-nitrobenzoic acid) (DTNB), the reaction mixture was incubated at 37 °C during 60 min and a new value (A_1_) was determined. The thiol groups concentration was calculated from A_1_ to A_0_ subtraction using a molar extinction coefficient of 13.6 × 10^3^ M^−1^ · cm^−1^. The results were expressed as nmol of thiol groups *per* mg of protein.

### H_2_O_2_ determination

The gastric mucosa H_2_O_2_ level was performed according to Dingeon et al. [28]. Briefly, the hydrogen peroxide reacts with p-hydroxybenzoic acid and 4-aminoantipyrine in the presence of peroxidase leading to the formation of quinoneimine that has a pink color detected at 505 nm.

### Antioxidant enzyme activity assays

The activity of SOD was determined by using modified epinephrine assays [29]. At alkaline pH, superoxide anion O_2_^−^ causes the autoxidation of epinephrine to adenochrome; while competing with this reaction, SOD decreased the adenochrome formation. One unit of SOD is defined as the amount of the extract that inhibits the rate of adenochrome formation by 50 %. Enzyme extract was added in 2 ml reaction mixture containing 10 μL of bovine catalase (0.4 U/μl), 20 μL epinephrine (5 mg/ml) and 62.5 mM sodium carbonate/bicarbonate buffer pH 10.2. Changes in absorbance were recorded at 480 nm.

The activity of CAT was assessed by measuring the initial rate of H_2_O_2_ disappearance at 240 nm [30]. The reaction mixture contained 33 mM H_2_O_2_ in 50 mM phosphate buffer pH 7.0 and the activity of CAT was calculated by using the extinction coefficient of 40 mM^−1^ cm^−1^ for H_2_O_2_.

The activity of GPx was quantified by the procedure of Flohé and Günzler [31]. Briefly, 1 mL of reaction mixture containing 0.2 mL of gastric mucosa supernatant, 0.2 mL of phosphate buffer 0.1 M pH 7.4, 0.2 mL of GSH (4 mM) and 0.4 mL of H_2_O_2_ (5 mM) was incubated at 37 °C for 1 min and the reaction was stopped by the addition of 0.5 mL TCA (5 %, w/v). After centrifugation at 1500 *g* for 5 min, aliquot (0.2 mL) from supernatant was combined with 0.5 mL of phosphate buffer 0.1 M pH 7.4 and 0.5 mL DTNB (10 mM) and absorbance was read at 412 nm. The activity of GPx was expressed as nmol of GSH consumed/min/mg protein.

### Statistical analysis

The data were analyzed by one-way analysis of variance (ANOVA) and were expressed as means ± standard error of the mean (S.E.M.). The data are representative of 10 independent experiments. All statistical tests were two-tailed, and a *p* value of 0.05 or less was considered significant.

## Results

### Acute oral toxicity of CPAE

In the acute oral toxicity study, neither abnormal behavior nor mortality was detected during the observation period. Thus, the LD50 value was greater than 20 g/kg b.w. for the aqueous extract of carob pods.

### In vitro DPPH and ABTS radical scavenging activities

Several concentrations ranging from 0–200 μg/ml of the CPAE were tested for their antioxidant activities in different *in vitro* models. We have found that the radical-scavenging activity of CPAE against DPPH and ABTS radicals increased significantly in a dose-dependent manner. The EC_50_ values calculated from the graph demonstrated that the RSA of CPAE (EC_50_ = 228.22 ± 5.27 μg/mL and 184.41 ± 3.95 μg/mL respectively for DPPH and ABTS radical-scavenging activity) appeared similar to that of ascorbic acid (EC_50_ = 190.47 ± 1.2 and 174.13 ± 0.9 μg/mL) as well known reference molecule (Table 1).

**Table 1:** EC_50_ values of DPPH and ABTS radical-scavenging activity carob pods aqueous extract (CPAE). EC_50_: the effective concentration of sample that can decrease DPPH or ABTS concentration by 50 %

	EC_50_ of DPPH radical-scavenging activity (μg/ml)	EC_50_ of ABTS radical-scavenging activity (μg/ml)
CPAE	228.22 ± 5.27	184.41 ± 3.95
Ascorbic acid	190.47 ± 1.2	174.13 ± 0.9

### Effect of CPAE on EtOH-induced acute macroscopic gastric injury and volume change

**Fig. 1:**
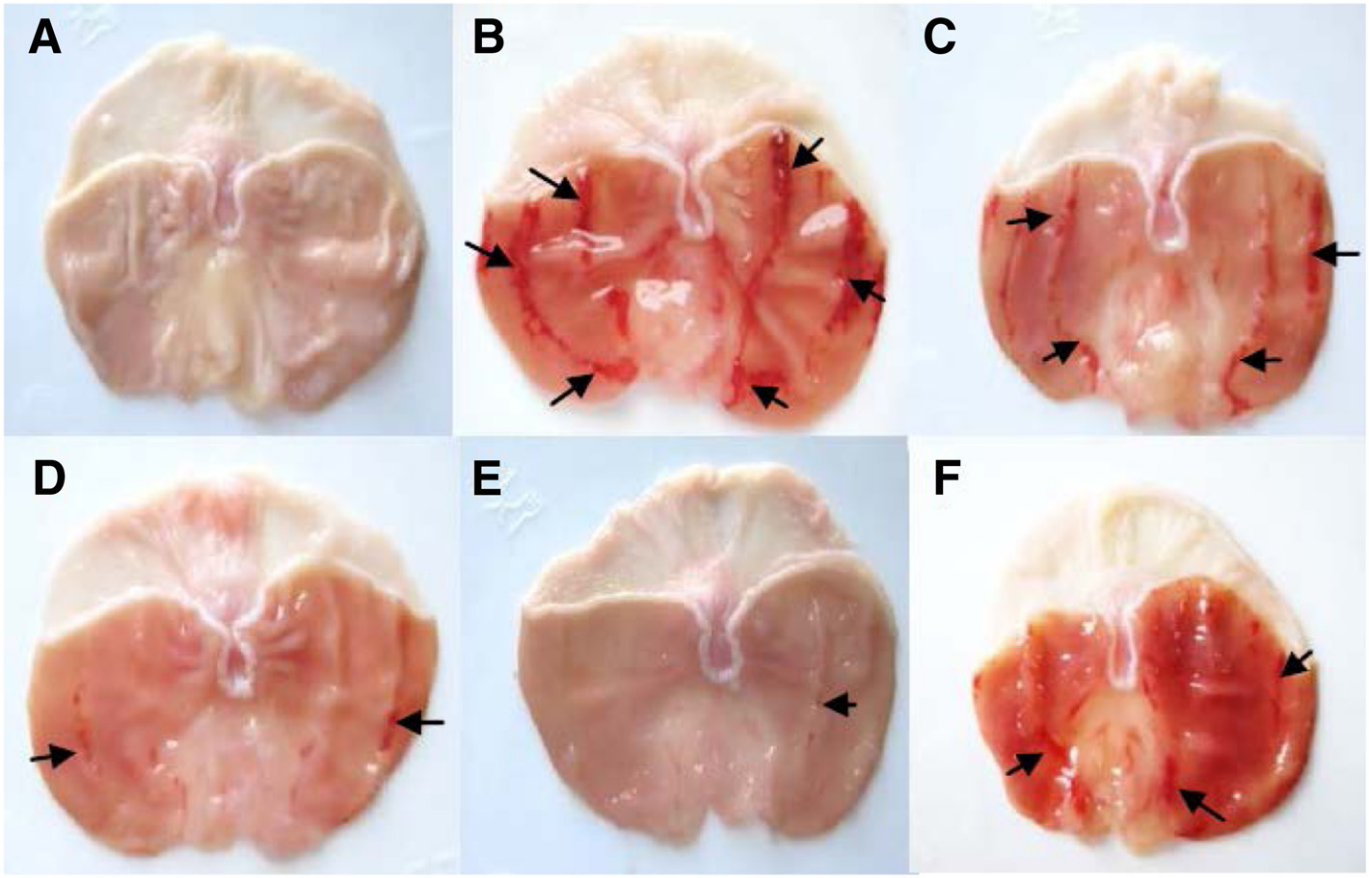
Subacute effect of caob pods aqueous extract (CPAE) and famotidine (FAM) on macroscopic changes induced by ethanol (EtOH) in rats. Animals were pre-treated with various doses of CPAE (500, 1000 and 2000 mg/kg, *b.w., p.o.*), FAM (10 mg/kg, *b.w., p.o.*) or bi-distilled water, challenged with a single oral administration of EtOH (4 g/kg, *b.w., p.o.*) or NaCl 9‰ for two hours. **a**: control; **b**: EtOH; **c**: EtOH+ CPAE-500; **d**: EtOH+ CPAE-1000; **e**: EtOH+ CPAE-2000 and **f**: EtOH+ FAM)

The macroscopic examination of gastric mucosa is shown in Fig. 1. As expected, EtOH administration exhibited injuries, including hemorrhage and hyperemia. CPAE and famotidine treatment showed a dose-dependent decrease in all macroscopic toxic signs compared with the EtOH treated group. Moreover, quantitative analysis showed that carob extract or reference molecule pre-treatment significantly and dose-dependently reduced the ulcer index, protected against the gastric volume juice decrease, and ameliorated the protection percentage of injury induced by EtOH administration (Table 2).

**Table 2:** Subacute effect of carob pods aqueous extract (CPAE), famotidine (FAM) and ascorbic acid (AA) on macroscopic quantitative changes induced by EtOH in rats: ulcer mucus volume, ulcer area and percentage protection. Animals were pre-treated with various doses of CPAE (500, 1000 and 2000 mg/kg, *b.w., p.o.*), FAM (10 mg/kg, *b.w., p.o.*) or bi-distilled water, challenged with a single oral administration of EtOH (4 g/kg, *b.w., p.o.*) or NaCl 9‰ for two hours

Group	Mucus volume (ml)	Ulcer index (mm^2^)	Protection percentage (%)
Control	4.3 ± 0.20	----	----
EtOH	1.9 ± 0.3^*^	86.2 ± 2.6^*^	00
EtOH + CPAE-500	2.9 ± 0	67.0 ± 3.6^#^	23.4
EtOH + CPAE-1000	3.4 ± 0.2^#^	16.6 ± 1.4^#^	79.8
EtOH + CPAE-2000	3.9 ± 0.3^#^	06.4 ± 0.9^#^	92.2
EtOH + FAM	3.8 ± 0.2^#^	26.2 ± 3.3^#^	68.1

*: *p* < 0.05 compared to control group and #: *p* < 0.05 compared to EtOH group

### Effect of CPAE on EtOH-induced gastric microscopic injury

**Fig. 2:**
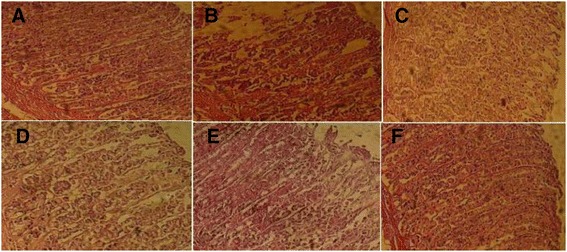
Subacute effect of caob pods aqueous extract (CPAE) and famotidine (FAM) on histological changes induced by ethanol (EtOH) in rats. Animals were pre-treated with various doses of CPAE (500, 1000 and 2000 mg/kg, *b.w., p.o.*), FAM (10 mg/kg, *b.w., p.o.*) or bi-distilled water, challenged with a single oral administration of EtOH (4 g/kg, *b.w., p.o.*) or NaCl 9‰ for two hours. **a**: control; **b**: EtOH; **c**: EtOH+ CPAE-500; **d**: EtOH+ CPAE-1000; **e**: EtOH+ CPAE-2000 and **f**: EtOH+ FAM)

We also examined the effect of EtOH and CPAE on gastric mucosa histology and the results are shown in Fig. 2. EtOH 80 % induced a marked erosive lesion in the gastric tissue. CPAE or famotidine pre-treatment greatly reduced the histopathological changes induced by acute alcohol intoxication.

### Effect of CPAE on EtOH-induced gastric lipoperoxidation and hydrogen peroxide increase

**Fig. 3:**
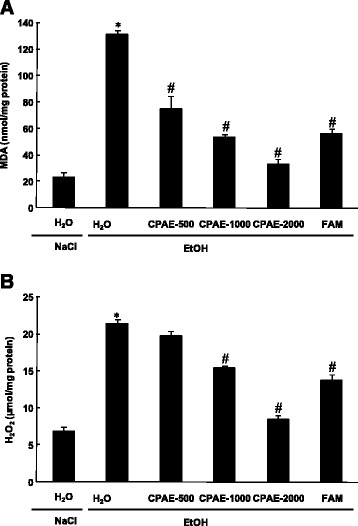
Subacute effect of caob pods aqueous extract (CPAE) and famotidine (FAM) ethanol (EtOH)-induced changes in stomach mucosa MDA **a** and H_2_O_2_ **b** levels in rats. in rats. Animals were pre-treated with various doses of CPAE (500, 1000 and 2000 mg/kg, *b.w., p.o.*), FAM (10 mg/kg, *b.w., p.o.*) or bi-distilled water, challenged with a single oral administration of EtOH (4 g/kg, *b.w., p.o.*) or NaCl 9‰ for two hours. *: *p* < 0.05 compared to control group and #: *p* < 0.05 compared to EtOH group

Bearing on the effect of EtOH and CPAE on oxidative stress condition, we firstly studied the gastric lipoperoxidation and hydrogen peroxide content (Fig. 3a). EtOH intoxication drastically increased the gastric MDA and H_2_O_2_ levels (Fig. 3b). CPAE pre-treatment significantly and dose-dependently reversed lipoperoxidation and hydrogen peroxide increase induced by EtOH intoxication.

### Effect of of CPAE on EtOH-induced gastric -SH groups decrease

**Fig. 4:**
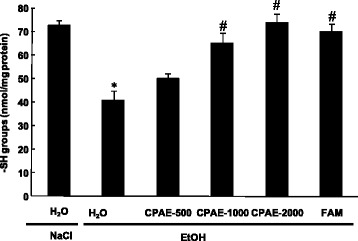
Subacute effect of caob pods aqueous extract (CPAE) and famotidine (FAM) ethanol (EtOH)-induced changes in stomach mucosa SH- groups level in rats. in rats. Animals were pre-treated with various doses of CPAE (500, 1000 and 2000 mg/kg, *b.w., p.o.*), FAM (10 mg/kg, *b.w., p.o.*) or bi-distilled water, challenged with a single oral administration of EtOH (4 g/kg, *b.w., p.o.*) or NaCl 9‰ for two hours. *: *p* < 0.05 compared to control group and #: *p* < 0.05 compared to EtOH group

We also showed that thiol groups level was significantly reduced in the gastric mucosa of alcohol-treated rats. However CPAE (500, 1000 and 2000 mg/kg, *b.w. p.o.*) or famotidine (10 mg/kg, *b.w. p.o.*) pre-treatment significantly protected against this decrease as compared to EtOH group (Fig. 4).

### Effect of CPAE on EtOH-induced antioxidant enzyme activities depletion

**Fig. 5:**
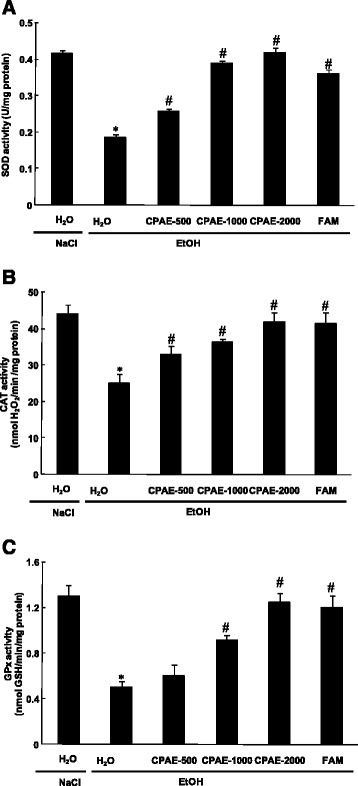
Subacute effect of caob pods aqueous extract (CPAE) and famotidine (FAM) ethanol (EtOH)-induced changes in stomach mucosa antioxidant enzyme activities : SOD **a**, CAT **b** and GPx **c** in rats.. in rats. Animals were pre-treated with various doses of CPAE (500, 1000 and 2000 mg/kg, b.w., p.o.), FAM (10 mg/kg, b.w., p.o.) or bi-distilled water, challenged with a single oral administration of EtOH (4 g/kg, *b.w., p.o.*) or NaCl 9‰ for two hours. *: *p* < 0.05 compared to control group and #: *p* < 0.05 compared to EtOH group

We further looked at the effect of EtOH and CPAE on antioxidant enzymes activities in gastric mucosa (Fig. 5). EtOH 80 % significantly increased stomach mucosa antioxidant enzyme activities as SOD (A) and CAT (B) but it significantly decreased the GPx activity (C). However, sub-acute pre-treatment with carob extract or famotidine significantly reduced the EtOH-induced increase and a decrease in antioxidant enzyme activities to near control levels with the highest dose.

## Discussion

The stomach is a sensitive digestive organ mainly exposed to exogenous pathogens from the diet. In response to these pathogens, the stomach tissue produces ROS such as hydroxyl radical a superoxide anion, which might be related to the development of gastric organic disorders like gastritis, gastric ulcers, and gastric cancer, as well as functional disorders such as functional dyspepsia [32]. Ethanol is considered one of the agents that induce gastric ulcers. The effects of ethanol on gastric mucosa are complicated and multifaceted that may be associated with a disturbance in the balance between gastric mucosal protective and aggressive factors [33]. Ethanol causes injures in the vascular endothelial cells of the gastric mucosa and induces microcirculatory disturbance and hypoxia, linking to the overproduction of oxygen radicals [34]. ROS are produced within the gastrointestinal tract, but their roles in pathophysiology and disease pathogenesis have not been well studied.

Many medicinal plants show in their chemical constitution, flavonoids, triterpenoids and tannins, which protect the stomach mucosa through the induction of gastroprotective mechanisms or acting as natural antioxidants [35–37]. Flavonoids and tannins are the major group of phenolic compounds that act as primary antioxidants or free radical scavengers [38].

Our phytochemical study, firstly, revealed that its richness total polyphenols, total flavonoids, and condensed tannins [14]. On the other hand, using the DPPH and ABTS radical-scavenging assay, we found that CPAE presents a high scavenging capacity, albeit lesser than ascorbic acid which was used as a reference molecule. The antioxidant capacity of carob is mainly related to the higher level of phenolic compouds in this fraction [14]. However, these molecules are the major source of their capacity of scavenging free radicals such as superoxide anion (O_2_^.^) and hydroxyl radical (OH^.^) [39].

*In vivo,* we firstly showed that alcohol administration provoked a clear macroscopic injuries, including hemorrhage and hyperemia as well as histopatological changes such as erosive lesions. CPAE pre-treatment significantly reversed EtOH-induced gastric mucosa macro- and microscopic lesions in a dose-dependent manner. However, gastric mucosa was previously shown to play a critical role in the protection of gastric barriers [40]. It is the first line of defense against acid and adheres together with bicarbonate secreted by the epithelium to serve as a barrier against self-digestion [41]. In addition, Gastric mucosa is an important protective factor for the gastric mucosa and is capable of acting as an antioxidant agent and reducing mucosal damage mediated by ROS [42]. Tannins could prevent ulcer development either via vasoconstricting effects, or due to their proteins-precipitating where it promotes precipitation of microproteins in the ulceration site, forming an impervious layer over the lining that hinders gut secretions and protects the underlying mucosa from irritants [43]. Moreover, flavonoids have anti-ulcer and gastroprotective properties [44]. However, EtOH-induced gastric ulceration has been previously shown to be attenuated by many plants extracts *Aqeratum conyzoides* [45], *Bacopa monniera and Azadirachta indica* [46], *Hippocratea excels* [47] and *Azadirachta indica* [48]. However, as far as we know, our report is the first one to deal with fruit extract of *Ceratonia siliqua* (carob) protective effect on acute EtOH-induced ulceration in rat gastric mucosa.

We also showed in the present study that EtOH intoxication induced lipid peroxidation, decrease of thiol groups level, increase of hydrogen peroxide content as well as depletion antioxidant enzyme activities such as SOD, CAT and GPx. Acute alcohol-induced oxidative stress was widely documented in gastric mucosa [49], liver [50], kidney [51], heart [52] and brain [53]. Ethanol administration provoked oxidative imbalance through a number of pathways including the generation of reactive oxygen species [54]. Lipid peroxidation level is an indicator of the generation of ROS in the tissue. However, SOD converts the reactive superoxide radical to H_2_O_2_, which was increased in the gastric mucosa and if not scavenged by CAT, it can by itself cause lipid peroxidation by generation of hydroxyl radical [55].

More importantly, we showed that carob extract pre-treatment abolished acute EtOH-induced oxidative stress in the gastric mucosa. These data fully corroborated all previously reported *in vivo* [17] and *in vitro* [15] antioxidant and anti-inflammatory properties of carob. We also demonstrated in our previous report of Sebai et al. [14] that the aqueous extract of carob pods contains a good amount of total polyphenols, total flavonoïds and condensed tannins. These molecules are the primal source of the antioxidant ability of this plant, by scavenging free radicals as hydroxyl radical (OH•) which is the major cause of lipid peroxidation [56]. In addition, it is well known that sulfhydryls are in part involved in gastric cytoprotection [57] and also in the maintain of mucosal barrier integrity and scavenge free radicals formed due to the action of noxious agents [58].

## Conclusion

In conclusion, our data clearly demonstrate that CPAE exerts protective effects against acute ethanol-induced ulceration in the rat gastric mucosa, in part thanks to its antioxidant properties.

## Abbreviations

CPAE: Carob pods aqueous extract
Carob_500_: Carob 500 mg/kg
Carob_1000_: Carob 1000 mg/kg
Carob_2000_: Carob 2000 mg/kg
CAT: Catalase
GPx: Glutathione peroxidase
H_2_O_2_: Hydrogenperoxide
MDA: Malondialdehyde
Prot: Proeins
SHs: Sulfhydryl groups
SOD: Superoxide dismutase
